# Influence of saponin extracts on enteric methane emission and rumen fermentation: a meta-analysis of in vitro experiments

**DOI:** 10.1186/s12917-026-05487-8

**Published:** 2026-05-11

**Authors:** Yulianri Rizki Yanza, Muhammad Ariana Setiawan, Ainisyya Fitri, Ujang Hidayat Tanuwiria, Cecep Hidayat, Fitri Ramadhani, Rahmat Hidayat, Rusli Fidriyanto, Rakhmad Perkasa Harahap, Vincent Niderkorn, Adib Norma Respati, Anuraga Jayanegara, Segun Abraham Olorunlowu, Julia Lagoda, Adam Cieslak, Agung Irawan

**Affiliations:** 1 Department of Animal Nutrition and Feed Technology, Faculty of Animal Husbandry, Universitas Padjadjaran, Sumedang, West Java 45363 Indonesia; 2 Department of Animal Science, Faculty of Agriculture, Khon Kaen University, Khon Kaen, Khon Kaen 40002 Thailand; 3 Research Center for Applied Zoology, National Research and Innovation Agency (BRIN), Bogor, West Java 16911 Indonesia; 4 Research Center for Animal Husbandry, National Research and Innovation Agency (BRIN), Bogor, West Java 16911 Indonesia; 5 Department of Biology Education, Islamic University of Riau, Pekanbaru, Riau 28284 Indonesia; 6 Study Program of Animal Science, Faculty of Agriculture, Tanjungpura University, Pontianak, West Kalimantan 78124 Indonesia; 7 Université Clermont Auvergne, INRAE, VetAgro Sup, UMR 1213 Herbivores, Saint-Genès Champanelle, France; 8 Department of Feed Technology, Politeknik Negeri Jember, Jember, East Java 68121 Indonesia; 9 Department of Animal Nutrition and Feed Technology, Faculty of Animal Science, IPB University, Bogor, West Java 16680 Indonesia; 10 Department of Animal Nutrition, Faculty of Veterinary Medicine and Animal Science, Poznan University of Life Sciences, Poznan, Poland; 11 Present Address: Vocational School, Universitas Sebelas Maret, Surakarta, Central Java 57126 Indonesia

**Keywords:** Greenhouse gases, Methane mitigation, Meta-regression, Phytochemicals, Protozoa

## Abstract

**Background:**

Saponin extracts have been extensively studied as natural modifiers for the rumen. This meta-analysis aims to quantify the dose-response effects of saponin extract supplementation on rumen fermentation profiles, digestibility, microbial populations, and methane production under in vitro conditions.

**Results:**

A review of peer-reviewed in vitro studies from Scopus, PubMed, and Semantic Scholar identified 36 articles with 379 datapoints and 24 saponin sources. Meta-regression analysis found quadratic responses of the levels of saponin extract with dry matter degradability (DMD), gas production, reduced methane production, protozoa abundance, and. NH_3_-N concentration. Subgroup meta-analysis revealed that the effects of saponin crude extract on in vitro rumen fermentation were source-dependent, with significant differences across most parameters. Subgroup meta-analysis demonstrated that DMD reduced by 8.0–19.1% (*P* < 0.001), except *Kometa lucerne* which increased DMD by + 14.3% (*P* < 0.001), Total VFA increased with *Calendula officinalis* (+ 20.5%), *Knautia arvensis* (+ 9.2%), and tea saponin (+ 28.8%) with increased on propionate by selected saponin sources (+ 7.7% to + 46.9%). Methane production was consistently reduced by 11–40.6%, with the greatest reduction observed for *Sapindus mukorossi* (*P* < 0.001). Protozoa were largely unaffected except for a modest reduction (− 10.5%; *P* = 0.028) with ivy.

**Conclusions:**

Modulating effects of saponin extracts on rumen fermentation and methane production are dependent on the origins and dose-dependent manner, suggesting diverse phytochemical characteristics of saponin from different sources. Certain sources of saponin extracts with potential methane-reducing properties were identified such as *Sapindus mukorossi*,* Albizia lebbeck*, *Calendula officinalis*,* Chenopodium quinoa*,* Sapindus saporia*,* Saponaria officinalis*,* Saponaria vaccaria*, and *Sesbania grandiflora*. However, optimized levels remain to be determined for specific saponin sources along with their specific phytochemical characteristics and specific mechanism how they modulates rumen fermentation.

## Background

The livestock industry is a major contributor to global greenhouse gas (GHG) emissions, accounting for approximately 9–11% of total anthropogenic emissions, with enteric CH_4_ emission representing 50–60% of livestock-derived GHG emissions [1, 2]. This issue is expected to become more critical in the coming decades as demand for animal-derived foods continues to increase with global population growth. In particular, global demand for animal-derived protein is projected to double by 2050 [3]. Beyond its environmental implications, enteric CH_4_ also represents a substantial nutritional and economic inefficiency in ruminant production systems because CH_4_ formation results in a loss of approximately 2–12% of feed gross energy (GE), which would otherwise be available for productive purposes [4–6]. This energy loss reduces feed utilization efficiency by increasing maintenance energy demands and limiting the energy available for growth and reproduction, which may lower productivity and increase feed costs [7]. Therefore, reducing enteric CH_4_ emissions is not only a climate mitigation priority but also a practical strategy to improve production efficiency and sustainability in ruminant systems, to support global sustainable development goals (SDGs). In this context, dietary interventions that modulate rumen fermentation have attracted considerable attention as feasible, scalable approaches to address environmental and production-related challenges simultaneously.

Considerable research has focused on manipulating rumen fermentation to improve feed efficiency and suppress methanogenesis [8, 9]. Among the available dietary strategies, plant secondary metabolites (PSM) have received increasing attention due to their ability to alter rumen microbial activity, reduce enteric CH_4_ formation, and serve as potential alternatives to antibiotic feed additives without promoting microbial resistance, while also contributing positively to the quality of animal products [10–12]. Within the PSM group, saponins are among the most widely studied compounds because of their distinctive structural and biological properties. Saponins are glycosidic compounds composed of sugar moieties linked to triterpene or steroid aglycones and are broadly distributed in many tropical plant species [13]. Their amphiphilic nature enables direct interaction with ruminal microbes, particularly protozoa-associated microbial populations, thereby influencing fermentation pathways and methane synthesis [14]. Previous studies have also documented broader benefits of saponin supplementation on animal performance and health across livestock species [15, 16]. Furthermore, a meta-analysis of in vivo studies by Yanza et al. [14] confirmed that certain saponin extracts can improve average daily gain and milk production, indicating that saponins may have both environmental and productivity-related value. These findings support the growing interest in saponins as natural rumen modifiers with potential practical relevance for sustainable livestock production.

Despite this progress, evidence from in vitro studies specifically addressing the effects of saponin extracts on enteric CH_4_ mitigation and rumen fermentation remains inconsistent, which limits the formulation of clear recommendations. On the one hand, several studies reported favourable responses. For example, Jayanegara et al. [17] showed that saponin extract from *Sapindus rarak* fruit effectively reduced methane production by decreasing protozoal populations and modifying the volatile fatty acid (VFA) profile, while Singh et al. [18] observed a linear decline in methane production with increasing concentrations of *Sapindus mucorossi* saponin extracts, suggesting a dose-dependent mitigation effect. On the other hand, Zhang et al. [16] reported contrasting findings, finding that tea saponin supplementation had no significant effect on methane production in an in vitro system. Such variation likely arises from multiple interacting factors, including the botanical source and chemical type of saponin, extraction form, dosage level, incubation conditions, and differences in rumen inoculum source and microbial composition. These interacting factors may alter fermentation dynamics and methanogenic responses in ways that are not directly comparable across individual experiments. Although the in vivo meta-analysis by Yanza et al. [14] provided important evidence at the animal level, a dedicated quantitative synthesis of in vitro experiments is still needed to clarify the direct fermentative and methanogenic responses under controlled conditions. The novelty of the present study lies in its specific focus on in vitro evidence and in its integrative evaluation of how saponin dose, source, and form shape rumen fermentation, digestibility, and methane outcomes across experiments, thereby addressing a key methodological and mechanistic gap in the existing literature.

Given the above, a meta-analytic framework is particularly appropriate for synthesizing the diverse and sometimes contradictory in vitro evidence, quantifying overall effect sizes, and identifying consistent response patterns across studies. By statistically integrating results from independent experiments, meta-analysis can provide a more robust estimate of the global effects of saponin extracts while accounting for between-study variability and potential moderators. This approach is expected to strengthen the evidence base for the use of saponins as natural methane-mitigating agents (SDGs 13) and to support more precise interpretation of their effects on the rumen ecosystem. Therefore, this study aimed to evaluate the effects of inclusion of saponins on in vitro rumen fermentation systems and identify the divergent effects of various doses, sources, and forms on rumen fermentation profiles, digestibility, and enteric CH_4_ production.

## Methods

### Database development

A structured database was compiled from peer-reviewed studies reporting in vitro rumen incubations in which saponin extracts were added to basal feed substrates and rumen fermentation responses were quantified. Relevant publications were identified through searches in Scopus, PubMed, and Semantic Scholar using combinations of the keywords *“saponin extract”*, *“ruminant”*, *“rumen fermentation”*, and *“in vitro”*. Studies were eligible for inclusion if they: (i) incorporated extracted saponins into the incubated substrate; (ii) provided a clear description of an in vitro rumen fermentation system using rumen fluid obtained from ruminants; (iii) were published in English; and (iv) reported at least one of the targeted outcomes, namely rumen fermentation traits, digestibility indices, rumen microbial population, methane, and/or total gas production.

### Data extraction

Data extraction was conducted by three researchers to minimize bias, following recommendations for independent review [19]. All relevant information from selected studies was compiled into Microsoft Excel, including study characteristics (authors, year, country), animal donors, basal substrate, in vitro methods, experimental design (treatments, inclusion levels, duration, diet composition), and response variables. Mean values and variability (SEM or converted from SD) were recorded for all measured parameters. Data presented graphically were digitized using WebPlotDigitizer. Quality control was ensured through random cross-checking of ~ 20% of entries by additional reviewers. Categorization was made for source of saponin extract, form of saponin, in vitro method, animal donor, and basal substrate. Individual experiments within studies were treated as separate observations. A weighting factor based on normalized inverse SEM was calculated for each outcome.

The final database comprised 379 datapoint from 36 articles and encompassed approximately 24 botanical sources of saponin extracts, including *Yucca schidigera*, *Quillaja saponaria*, *Acacia auriculoformis*, *Trigonella foenum-graecum*, *Carduus pycnocephalus*, *Sesbania sesban*, *Knautia arvensis*, Quillaja bark, *Saponaria officinalis*, *Calendula officinalis*, *Gypsophila paniculata*, *Medicago sativa*, *Argania spinosa*, *Saponaria vaccaria* L., *Chenopodium quinoa*, *Primula veris*, *Albizia lebbeck*, ivy, *Sapindus saponaria*, tea saponin, *Sapindus mucorossi*, *Sesbania grandiflora*, *Mucuna pruriens*, Verko lucerne, and Kometa lucerne. Extracts were obtained from multiple plant parts (e.g., fruits, seeds, floral heads, leaves, wood, and roots). The primary outcomes of interest were the rumen fermentation profile and CH₄ production.

Prior to statistical analyses, supplementation doses and response variables were harmonised to ensure comparability across studies. Saponin inclusion level was standardised as g/kg DM. Fermentation outcomes were converted to common units, with total VFA expressed as mmol/L and ruminal ammonia as mg/dL. Individual VFA were expressed on a molar basis (e.g., mol/100 mol). Digestibility outcomes (DMD and OMD) were standardised to consistent units (e.g., g/100 g or %), microbial counts (bacteria and protozoa) were converted to log10/mL, and gas outputs (total gas production and methane) were expressed as mL, mL/g DM, and mL/g degraded DM as appropriate.

### Data analysis

Meta-regression analysis was conducted using SAS version 9.4 following a mixed-model approach implemented in PROC MIXED. The method followed established methodology for animal nutrition meta-analyses [20]. Consistent with approaches adapted from previous rumen meta-analyses [14, 21], levels of crude extract saponin was modelled as a fixed effect and evaluated using linear and quadratic dose–response terms, while between-study/trial variation was accounted for through random effects. The general model was:

$$\begin{aligned}\:{Y}_{ij}&=\mu\:+{s}_{i}+{\tau\:}_{j}+s{\tau\:}_{ij}\\&+{B}_{0}+{B}_{1}{X}_{ij}+{B}_{2}{X}_{ij}^{2}\\&+{B}_{i}{X}_{ij}+{e}_{ij}\end{aligned}$$

where $\:{Y}_{ij}$is the dependent variable, $\:\mu\:$is the overall mean, $\:{s}_{i}$is the random effect of the $\:i$th trial, $\:{\tau\:}_{j}$is the fixed effect of the $\:j$th level factor, $\:s{\tau\:}_{ij}$represents the random interaction between trial and the $\:j$th factor level, $\:{B}_{0}$is the overall intercept (fixed), $\:{B}_{1}$and $\:{B}_{2}$are the fixed linear and quadratic coefficients of the response on dose ($\:{X}_{ij}$), $\:{B}_{i}{X}_{ij}$allows the slope to vary by study when applicable, and $\:{e}_{ij}$is the residual error term. The number of replicates was incorporated as a weighting factor using the SAS WEIGHT statement. Statistical significance was declared at *P* < 0.05, and trends were discussed at 0.05 < *P* < 0.10.

To examine differences among botanical sources of saponins extract, plant origin was treated as a categorical fixed effect. The sources or origins included *Yucca schidigera*, *Quillaja saponaria*, *Acacia auriculoformis*, *Trigonella foenum-graecum*, *Carduus pycnocephalus*, *Sesbania sesban*, *Knautia arvensis*, Quillaja bark, *Saponaria officinalis*, *Calendula officinalis*, *Gypsophila paniculata*, *Medicago sativa*, *Argania spinosa*, *Saponaria vaccaria* L., *Chenopodium quinoa*, *Primula veris*, *Albizia lebbeck*, ivy, *Sapindus saponaria*, tea saponin, *Sapindus mucorossi*, *Sesbania grandiflora*, and *Mucuna pruriens*. The analysis of categorical variables were carried out using multilevel subgroup meta-analysis via “*metafor*” package [22] in RStudio. For this purpose, we calculated the effect size using weighted relative mean difference (RMD), which is defined as % change from treatment groups (category) compared to control substrate, adjusted by inverse variance (standard of errors; SE) as weighting factor. This approach allows direct and consistent interpretations across different units of measurement and types of datasets [23]. In many studies, non-independent study effect existed due to shared similar control group within supplementary levels. This was handled using a multilevel meta-analysis approach with the *rma.mv()* function in the metafor package, as previously suggested [24]. This approach modelled hierarchical random-effects structure in which individual comparisons were nested within studies to appropriately account for within-study correlations from shared control groups. The mixed effect models were fitted using both univariate “rma” and multivariate “rma.mv” functions with a Maximum Likelihood estimator (type = ML) and intercept-free structure. In addition, heterogeneity raised from between-study variance was calculated using Cochran’s Q and *I* statistics [25]. Egger test was carried out to identify significance of publication bias.

## Results

### Datasets and descriptive statistics of response variables included in the database

**Fig. 1:**
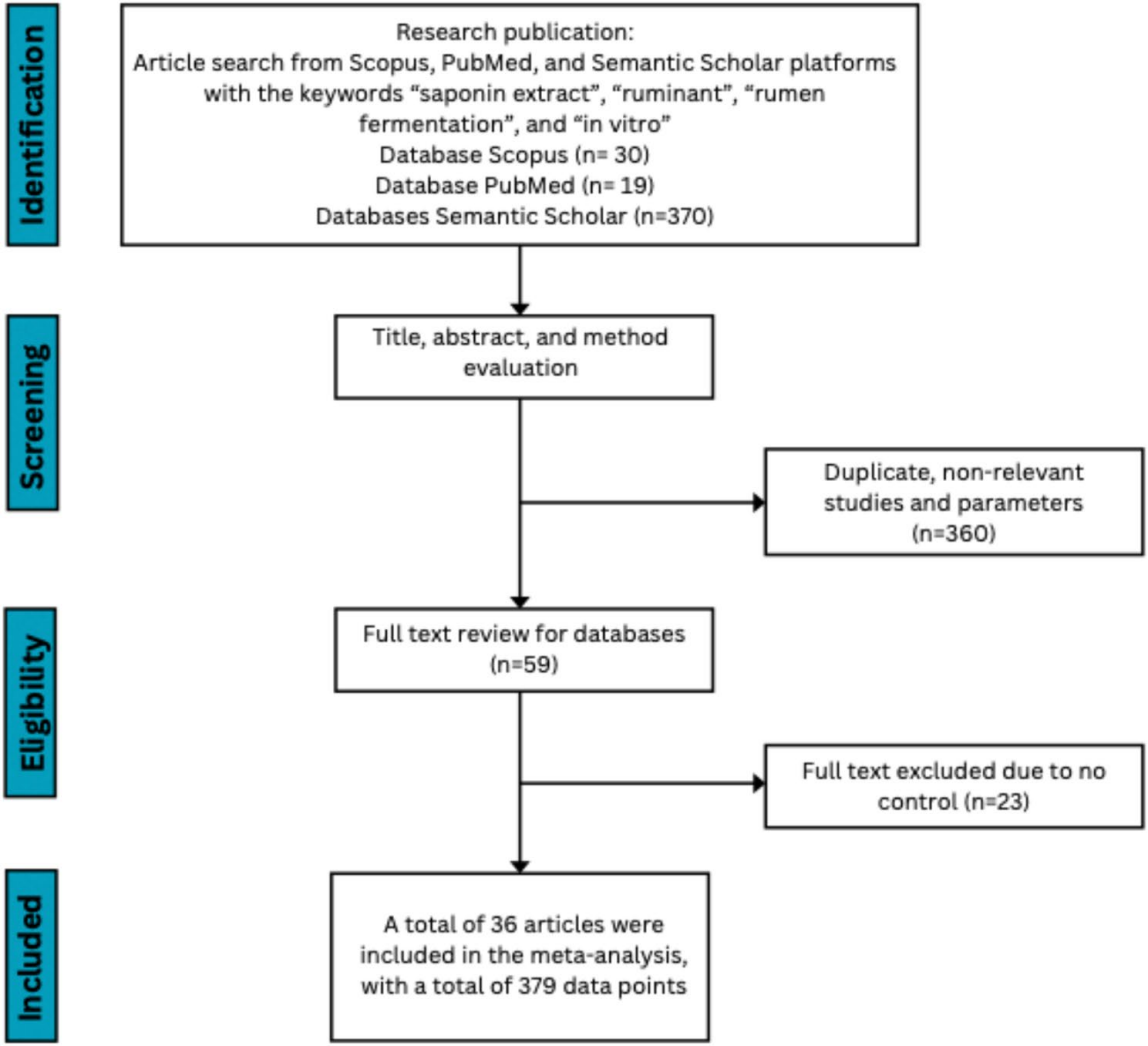
Diagram flow for selection of the studies on the influence of saponin extract based on PRISMA protocols

The literature search for this meta-analysis identified 36 studies (Fig. 1) that investigated the supplementation of ruminant feed substrates with saponin extracts, all tested in vitro. This search also collected information on the preservation methods (liquid or powder) and the sources of the saponin extracts, as shown in Table 1. A total of 27 sources of saponins were examined in vitro, with *Yucca schidigera* (YS) being the most used source for saponin extraction (31.1% of the dataset).

**Table 1:** List of studies and their characteristics used in the meta-analysis

No	References	Methods	Diet	Saponin Source	Form	Saponin Extract Level	
						g/kg DM	mL/L
1	Valdez et al. [26]	RST	Concentrate	YS	Powder	0–77	
2	Makkar et al. [27]	HGT	Hay, Concentrate	YS, QS, AA	Powder	0-180	
3	Wang et al. [28]	RST	Alfafa hay, Concentrate	YS	Powder	0-40.75	
4	Wang et al. [29]	BC	Barley grain, Barley silage	YS	Powder	0-22.5	
5	Sliwinski et al. [30]	RST	Forage	YS	Powder	0–8	
6	Lila et al. [31]	BC	Hay, Concentrate	YS	Powder	0-480	
7	Hristov et al. [32]	RST	Alfafa hay, Grain, Soybean meal	YS	Powder	0–32	
8	Cardozo et al. [33]	BC	Forage, Concentrate	YS	Powder	0–30	
9	Busquet et al. [34]	BC	Forage, Concentrate	YS	Powder	0-300	
10	Pen et al. [35]	RST	Oat, Alfafa hay	YS, QS	Liquid		0-480
11	Pen et al. [36]	RST	Concentrate, Hay oat	QS	Liquid		0-240
12	Goel et al. [37]	HGT	Hay, concentrate	CP, TFG, SS, KA	Powder	0-35.43	
13	Lourenço et al. [38]	RST	Perennial ryegrass	QB	Powder	0-53.3	
14	Khiaosa-Ard et al. [39]	RST	Hay	YS	Powder	0-37.5	
15	Staerfl et al. [40]	HGT	Maize silage, Concentrate	YS	Powder	0-2.5	
16	Xu et al., [41]	BC	Alfafa hay, Fescue grass, Orchard grass, Bermuda grass, Switch grass, corn-based concentrate	YS	Powder	0-0.11	
17	Jayanegara et al. [42]	HGT	Hay, Concentrate	TFG, SS, KA	Powder	0-57.37	
18	Narvaez et al. [43]	BC	Barley	YS	Powder	0–65	
19	Budan et al. [44]	HGT	Roughage, Concentrate	Alfalfa, SO, CO, GP, TFG, YS, AS, SV, CQ, QS, QS, PV	Powder	0-17.59	
20	Yuliana et al. [45]	BC	Brachiaria humidicola grass, Indigofera sp	SR	Powder	0-1000	
21	Cieslak et al. [46]	BC	Meadow hay, Barley	SO	Powder	0-500	
22	Budan et al. [47]	HGT	Ray grass roughage, Wheat seeds	CO, SO	Powder	0–50	
23	Sirohi et al. [48]	BC	Roughage, Concentrate	AL	Powder	0–60	
24	Yogianto et al. [49]	BC	Forage, Concentrate	SR	Powder	0-200	
25	Belanche et al. [50]	BC	Alfalfa hay, Grass hay, Barley, SBM, Mineral, Vitamin	Ivy	Powder	0-200	
26	Galindo et al. [51]	BC	Star grass (Cyndon nlemfuensis)	Ssap	Powder	0–18	
27	Guyader et al. [52]	BC	Wheat, Corn	TS	Powder	0–50	
28	Ramos-Morales et al. [53]	BC	Ryegrass, Barley	IV	Powder	0-100	
29	Singh et al. [18]	HGT	Oats hay	SM	Liquid	-	0–10
30	Yuliana et al. [54]	HGT	Cassava leaf silage	SR, HS	Powder	0–40	
31	Kozłowska et al. [55]	HGT	-	VL, KL	Powder	3.3–41.6	
32	Jayanegara et al. [17]	BC	Forage, Concentrate	SR	Powder	0-1000	
33	Kozłowska et al. [56]	RST	Forage, Concentrate	VL, KL	Powder	0-0.01	
34	Unnawong et al. [57]	HGT	Roughage, Concentrate	SG	Powder	0–6	
35	Zhang et al. [16]	HGT	Corn, SBM, Alfafa hay, Wheat bran	TS	Powder	0–20	
36	Jin et al. [58]	BC	Guinea grass, Alfafa, concentrate	YS	Powder	0–24	

Rusitec, *BC* Batch culture, *HGT* Hohenheim gas test, *YS *Yucca schidigera, *QS* Quillaja saponaria, *AA *Acacia auriculoformis, *TFG *Trigonella foenum-graecum,* CP* Carduus pycnocephalus, *SR* Sapindus rarak, *HS *Hibiscus sp., *SS *Sesbania sesban, *KA *Knautia arvensis, *QB *Quillaja bark, *SO *Saponaria officinalis, *CO *Calendula officinalis, *GP *Gypsophila paniculata, *AS *Argania spinosa, *SV *Saponaria vaccaria L., *CQ *Chenopodium quinoa, *PV *Primula veris, *AL *Albizia lebbeck, *IV *ivy, *Ssap *Sapindus saporia, *TS* tea saponin, *SM *Sapindus mukorossi, *SG *Sesbania grandiflora, *MP *Mucuna pruriens, *VL *Verko lucerne, *KL *Kometa lucerne

Table 2 presents descriptive data outlining the ranges of measured parameters. The dataset covered indicators of digestibility, rumen fermentation profile, gas and methane production (including kinetics), and microbial parameters, with sample sizes ranging from *n* = 17 to 379 across variables. The variability may be attributed to differences in methods, donor animals, and the basal diets used in the studies.

**Table 2:** Descriptive statistics of the variables in the database used to evaluate the influence of saponin extract supplementation

Response variables	Unit	n	Min	Max	Mean	SEM
Digestibility						
DMD	g/kg	130	227.4	909.4	577.3	11.8
OMD	g/kg	41	429.9	842.9	664.8	19.1
Rumen Fermentation Profile						
pH		179	5.7	7.94	6.51	0.03
NH_3_-N	mg/dL	261	0.794	65.28	17.67	0.716
TVFA	mmol/L	260	24.34	1035.2	140.27	12.63
C_2_	mol/100 mol	203	34.1	75.2	59.92	0.585
C_3_	mol/100 mol	203	14.5	49.4	25.14	0.439
C_4_	mol/100 mol	203	5.257	18.6	10.81	0.210
IsoC_4_	mol/100 mol	96	0.45	6.03	1.63	0.102
C_5_	mol/100 mol	99	0.714	7.87	2.63	0.173
IsoC_5_	mol/100 mol	96	0.17	4.38	2.02	0.089
BCVFA	mol/100 mol	149	0.44	17.79	3.69	0.246
C_2_:C_3_		210	0.707	4.779	2.67	0.065
Total Gas and CH_4_ Production						
Gas Production	mL	197	2.49	5111	233.590	51.10
	mL/g DM	197	4.981	2726.3	307.74	31.31
	mL/g DM	78	16.13	4247.5	634.80	102.24
	mL/g OM	17	81.935	4359.1	1467.9	413.32
Gas kinetics						
a+b	mL	37	28.32	107.51	57.326	4.186
c	mL/h	41	0.03	0.85	0.092	0.020
CH_4_	mL	220	0.478	360	46.819	6.014

*DM* dry matter, *DMD* dry matter digestibility, *OMD* organic matter digestibility, *NH*_3_-N ammonium nitrogen, *TVFA* total volatile fatty acids, *C*_2_ acetate, *C*_3_ propionate, *C*_4_ butyrate, *IsoC*_4_ iso-butyrate, *C*_5_ valerate, *IsoC*_5_ iso-valerate, *BCVFA* branched-chain volatile fatty acids, *C*_2_:C_3_ acetate-to-propionate ratio, *a+b* potential extent of gas production, *c* rate constant of gas production of the insoluble fraction, *CH*_4_ methane, *CO*_2_ carbon dioxide, *H*_2_ hydrogen

### Dose–response meta-regression on response variables

The dose–response meta-regression analysis indicated that supplementation with saponin crude extract was significantly associated with changes in various in vitro digestibility and rumen fermentation variables. Notably, both linear (L) and quadratic (Q) patterns were observed across these outcomes (Table 3). Moreover, for several response variables, the effect of inclusion levels varied based on the source and/or form of saponin, as evidenced by significant interaction terms for dose × source and dose × form (Table 3).

**Table 3:** Dose-response meta-regression of the influence of saponin extract supplementation on in vitro digestibility, rumen fermentation profile, and enteric methane emission

Response variables	Unit	n	Model	Parameter estimates				Model statistics						
				Intercept	SE _Intercept_	Slope	SE _Slope_	P value		RMSE	AIC	D ´ Sources	D ´ Forms	
Digestibility														
DMD	g/kg	130	Q	598.1	23.02	-0.348	0.08	<0.001	37.23		1476.3	<0.001		0.499
						0.0003	0.00008	<0.001						
OMD	g/kg	41	Q	664	31.63	-0.39	0.15	0.016	32.98		471.5	0.520		0.543
						0.0004	0.0002	0.015						
Rumen Fermentation Profile														
pH		179	L	6.55	0.062	-0.0002	0.00005	0.0003	0.05		-228	<0.001		0.038
NH_3_-N	mg/dL	261	Q	17.61	1.36	-0.025	0.0042	<0.001	3.07		1661.3	0.050		0.104
						0.00004	0.000005	<0.001						
TVFA	mmol/L	260	L	108.9	23.8	0.011	0.014	0.430	19.00		2732.3	<0.001		0.820
C_2_	mol/100mol	203	Q	59.96	1.07	-0.017	0.0037	<0.001	2.40		1218.9	<0.001		0.001
						0.00001	0.000005	0.026						
C_3_	mol/100mol	203	Q	24.38	0.77	0.015	0.0038	0.0010	2.54		1193.7	<0.001		0.126
						-0.00001	0.000005	0.074						
C_4_	mol/100mol	203	Q	10.63	0.39	0.0025	0.0012	0.049	0.81		785.2	<0.001		<0.001
						-0.000003	0.000002	0.099						
IsoC_4_	mol/100mol	96	L	1.60	0.16	-0.0001	0.00036	0.773	0.44		235.4	0.295		0.886
C_5_	mol/100mol	99	Q	3.23	0.26	-0.0012	0.0007	0.076	0.33		277	0.980		0.532
	mol/100mol					0.000001	0	<0.001						
IsoC_5_	mol/100mol	96	L	2.24	0.15	-0.0004	0.0002	0.011	0.18		128.2	0.073		0.002
Total BCVFA	mol/100mol	149	L	3.71	0.45	-0.0009	0.0005	0.069	0.65		514.9	0.001		0.565
C_2_:C_3_		210	Q	2.70	0.12	-0.002	0.0005	<0.001	0.31		386.9	<0.001		0.032
						0.000001	0	<0.001						
Gas Production														
TGP	mL	197	L	332.5	100.00	-0.0006	0.019	0.973	30.40		2385.3	0.005		0.678
	mL/ g DM	197	L	301.0	61.20	0.012	0.020	0.548	32.39		2317.5	0.011		0.171
	mL/g DMD	78	Q	678.1	218.7	0.732	0.26	0.006	108.32		1083.9	0.100		0.090
						-0.0007	0.0003	0.015						
	mL/g OMD	17	Q	1106.3	549.5	12.72	3.39	0.005	167.84		253.4	0.985		-
						-0.045	0.017	0.025						
Gas Kinetic														
a+b	mL	37	L	60.35	10.04	-0.020	0.014	0.153	3.13		237.9	0.055		0.069
c	mL/h	41	L	0.099	0.037	0.000069	0.00036	0.850	0.10		-31	<0.001		0.973
Enteric CH_4_ production														
CH_4_	mL	220	Q	50.20	11.71	-0.043	0.012	0.004	8.27		1984	0.057		0.934
						0.000034	0.000013	0.013						
	mL/g DM	220	Q	48.70	10.39	-0.057	0.013	<0.001	9.30		2019.8	0.008		0.001
						0.00005	0.00002	0.002						
	mL/g DMD	43	L	239.12	99.379	-0.0107	0.028	0.704	28.70		452.8	0.806		0.231
CO_2_	mL	30	Q	908.89	268.61	1.87	0.69	0.013	104.95		412.4	0.877		0.975
						-0.0009	0.0014	0.538						
H_2_	mL	43	L	3.87	0.95	-0.0019	0.0005	0.001	0.42		117	0.459		-
Microbial Population														
Bacteria	Log/mL	53	L	8.03	0.70	0.000004	0.00052	0.993	0.64		193.3	0.994		0.749
Protozoa	Log/mL	148	Q	5.60	0.17	-0.00079	0.00022	0.001	0.13		80.6	<0.001		0.042
						0.00000028	0	<0.001						
MPS	mg/dL	32	Q	381.4	60.23	-11.27	3.24	0.002	66.47		387.1	-		0.006
						0.19	0.057	0.004						

*DM* dry matter, *DMD* dry matter digestibility, *OMD* organic matter digestibility, *NH*_3_-N ammonium nitrogen, *TVFA* total volatile fatty acids, *C*_2_ acetate, *C*_3_ propionate, *C*_4_ butyrate, *IsoC*_4_ iso-butyrate, *C*_5_ valerate, *IsoC*_5_ iso-valerate, *C*_2_:C_3_ acetate-to-propionate ratio, *BCVFA* branched-chain volatile fatty acids, *TGP* total gas production, *a+b* potential extent of gas production, *c* rate constant of gas production of the insoluble fraction, *CH*_4_ methane, *L* linear term, *Q* quadratic term, *n* sample size, *SE intercept* standard error of the intercept, *SE slope* standard error of the slope, *AIC* Akaike information criterion, *RMSE* root mean square error, *D × Form* interaction between dose and saponin form, *D × Sources* interaction between dose and saponin source

Regarding digestibility, saponin supplementation showed a significant negative dose–response relationship. Specifically, dry matter digestibility (DMD; *n* = 130) followed a quadratic trend, indicating a decrease in DMD as the dosage increased (β₁ = −0.348, *P* < 0.001), complemented by a significant polynomial term (β₂ = 0.000331, *P* < 0.001). Similarly, a quadratic response was observed for organic matter digestibility (OMD; *n* = 41), which decreased consistently with increasing dosage (β₁ = −0.39, *P* = 0.016), and this was further supported by a significant quadratic term (β₂ = 0.00038, *P* = 0.015).

Furthermore, rumen pH (*n* = 179) exhibited a small yet significant linear decline with increasing the levels of crude saponin supplementation (β₁ = −0.00018, *P* = 0.0003). Additionally, NH₃ concentration showed a substantial decrease in response to increasing crude saponin extract; both NH₃ (*n* = 261) and NH₃-N (*n* = 261) demonstrated significant quadratic relationships (NH₃: β₁ = −0.031, *P* < 0.001; β₂ = 0.00003, *P* < 0.001; NH₃-N: β₁ = −0.025, *P* < 0.001; β₂ = 0.000024, *P* < 0.001). In contrast, total volatile fatty acids (VFA; *n* = 260) did not exhibit a significant association with levels of supplementation (β₁ = 0.011, *P* = 0.430).

Moreover, the molar proportions of VFA shifted with crude saponin extract supplementation. For instance, acetate (*n* = 203) significantly decreased with increasing saponin levels (β₁ = −0.017, *P* < 0.001) and displayed a small but significant quadratic pattern (β₂ = 0.000011, *P* = 0.026). Conversely, propionate (*n* = 203) increased with increased saponin levels (β₁ = 0.015, *P* = 0.001), while butyrate (*n* = 203) exhibited a modest positive association (β₁ = 0.0025, *P* = 0.049). On the other hand, iso-butyrate (*n* = 96) and valerate showed no relationship with saponin supplementary levels (*P* = 0.773 and 0.076, respectively). Iso-valerate (IsoC5; *n* = 96) decreased linearly with dosage (β₁ = −0.00041, *P* = 0.011). Additionally, total branched-chain VFA (*n* = 149) showed a tendency to decline, although it did not achieve statistical significance (*P* = 0.069). Consistent with the responses observed for acetate and propionate, the acetate/propionate ratio (*n* = 210) significantly decreased with dosage (β₁ = −0.0020, *P* < 0.001), and this was further supported by a significant quadratic term (β₂ = 0.0000014, *P* < 0.001).

Results on total gas production (TGP) measured in mL (*n* = 197) and mL/g DM substrate (*n* = 197) indicated no significant association with saponin levels (both *P* ≥ 0.548). However, when gas production was evaluated relative to degraded substrate, significant quadratic relationships were identified for mL/g degraded DM (*n* = 78; β₁ = 0.732, *P* = 0.006; β₂ = −0.00069, *P* = 0.015) and mL/g degraded OM (*n* = 17; β₁ = 12.72, *P* = 0.005; β₂ = −0.045, *P* = 0.025). On the other hand, gas kinetic parameters (a + b, *n* = 37; c, *n* = 41) were not impacted by saponin levels (both *P* ≥ 0.153).

Methane (CH₄) production decreased with increasing saponin crude extract levels following curvilinear pattern (β₁ = −0.043, *P* = 0.004; β₂ = 0.000034, *P* = 0.013). Furthermore, a more pronounced reduced effect was observed when CH₄ was expressed per g DM substrate (*n* = 220; β₁ = −0.057, *P* < 0.001; β₂ = 0.00005, *P* = 0.002); however, CH₄ per g degraded DM (*n* = 43) was not associated with dosage (*P* = 0.704). Conversely, CO₂ output (*n* = 30) increased with dosage (β₁ = 1.87, *P* = 0.013), while H₂ (*n* = 43) decreased linearly (β₁ = −0.0019, *P* = 0.001).

Furthermore, microbial counts showed varied responses. Notably, total bacteria (*n* = 53) did not show a relationship with dosage (*P* = 0.993). In contrast, protozoa (*n* = 148) exhibited a significant decline, characterised by a quadratic relationship (β₁ = −0.00079, *P* = 0.001; β₂ = 0.00000028, *P* < 0.001). Additionally, microbial protein synthesis (MPS; *n* = 32) displayed a significant quadratic pattern with a negative linear component (β₁ = −11.27, *P* = 0.002) alongside a positive quadratic term (β₂ = 0.19, *P* = 0.004).

### Subgroup meta-analysis on major parameters of in vitro rumen fermentation

**Fig. 2:**
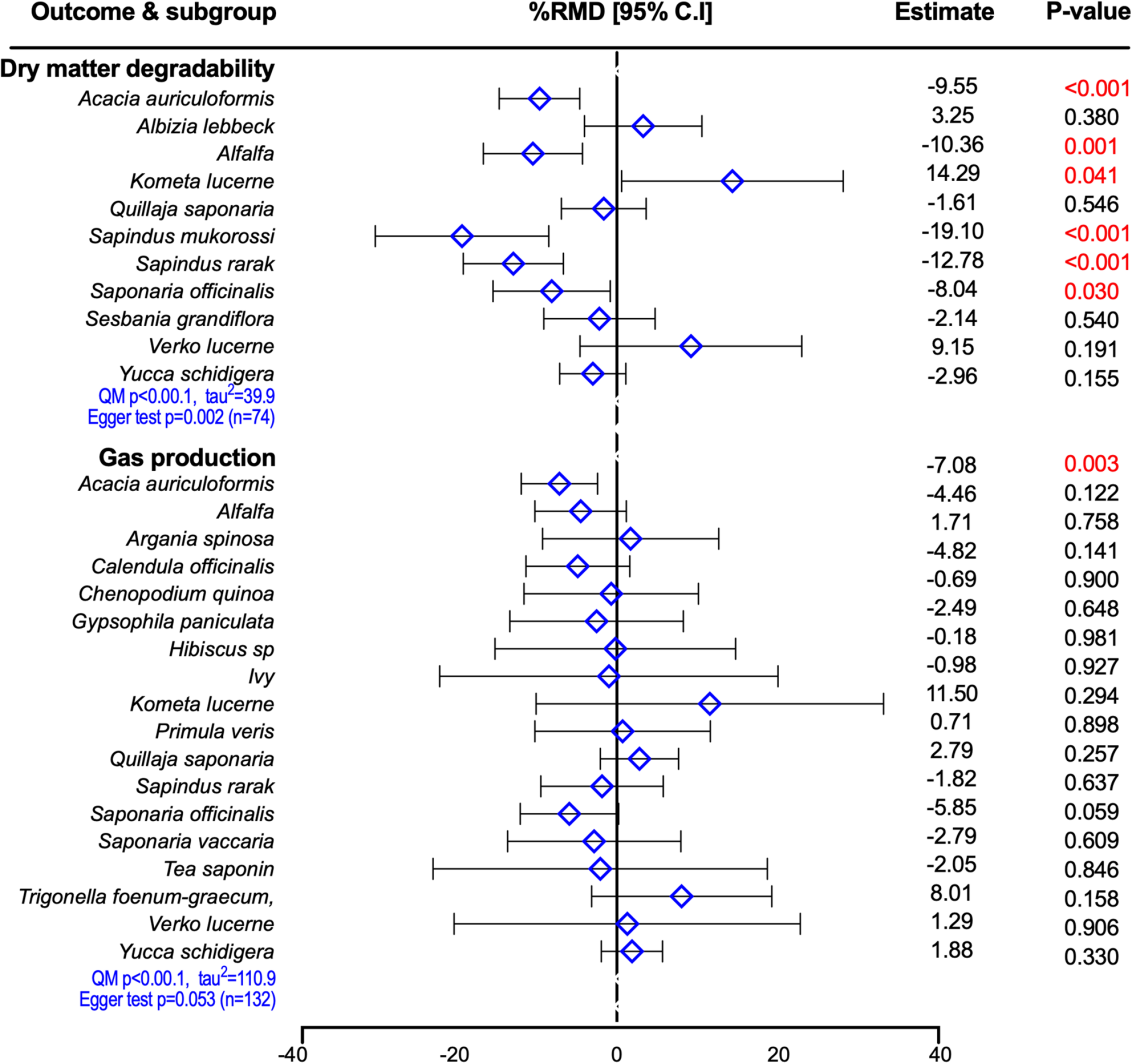
Multilevel subgroup meta-analysis of the effects of saponin sources on dry matter degradability (g/kg DM) and in vitro rumen gas production. The effect size is expressed at percentage change of weighted raw mean difference (%RMD) at 95% confidence intervals between treatment (saponin extract vs. control substrate). The central-black line represents the zero effect (RMD = 0); the blue-diamonds represent represents the %RMD of subgroup effect

**Fig. 3:**
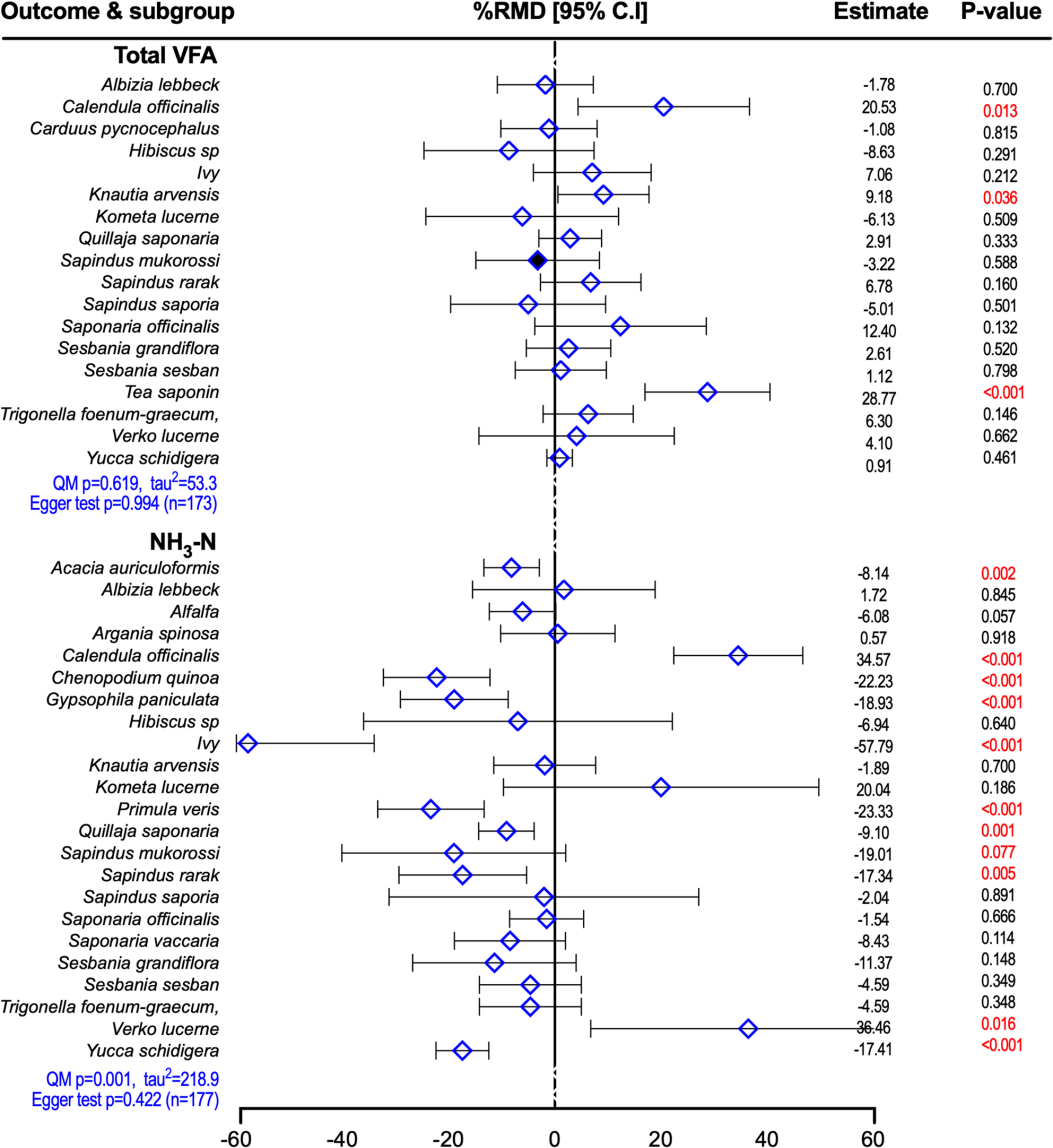
Multilevel subgroup meta-analysis of the effects of saponin sources on total volatile fatty acid (VFA; mmol) and NH_3_-N. The effect size is expressed at percentage change of weighted raw mean difference (%RMD) at 95% confidence intervals between treatment (saponin extract vs. control substrate). The central-black line represents the zero effect (RMD = 0); the blue-diamonds represent represents the %RMD of subgroup effect

**Fig. 4:**
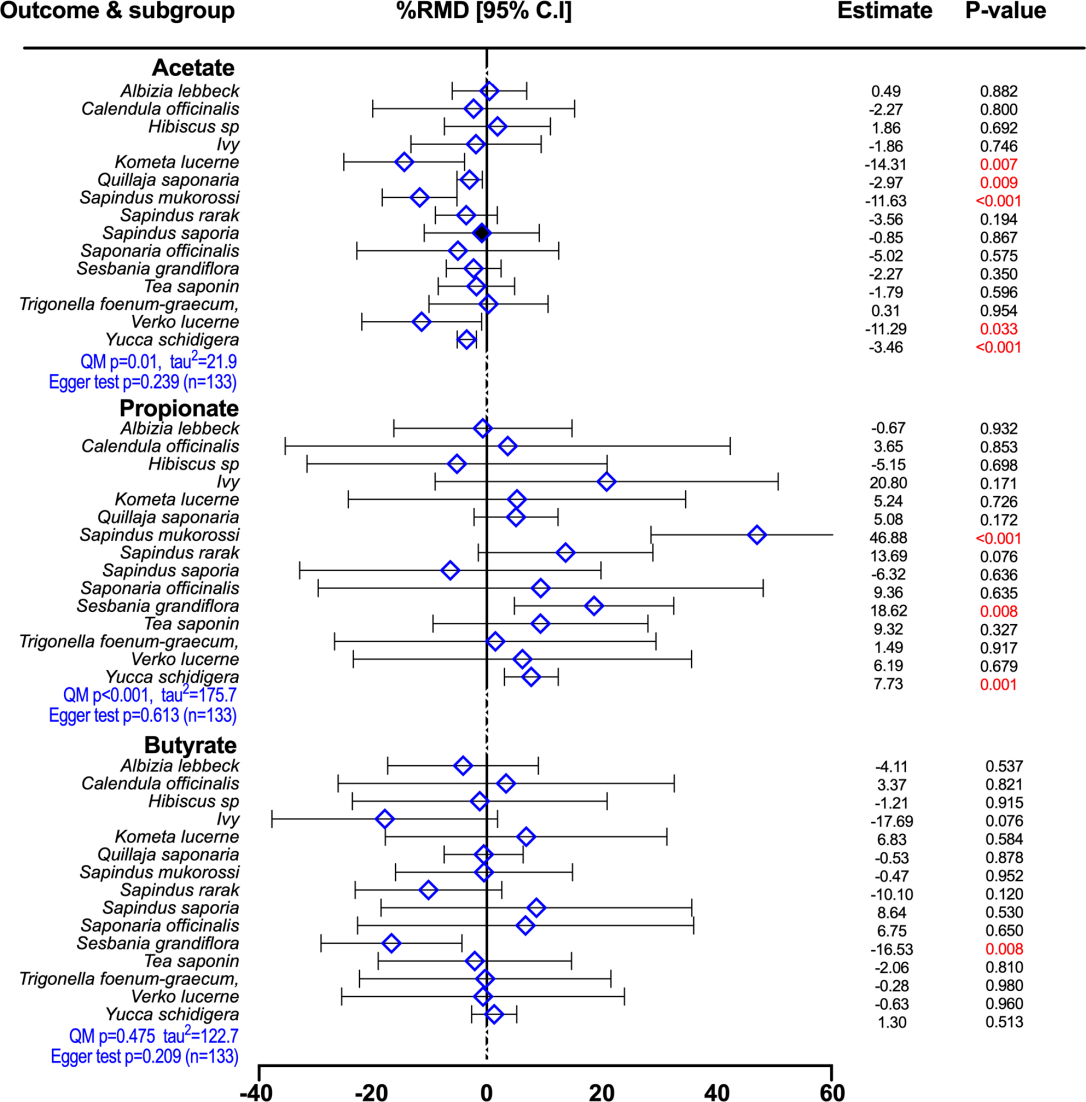
Multilevel subgroup meta-analysis of the effects of saponin sources on the proportion of individual VFA (acetate, propionate, butyrate). The effect size is expressed at percentage change of weighted raw mean difference (%RMD) at 95% confidence intervals between treatment (saponin extract vs. control substrate). The central-black line represents the zero effect (RMD = 0); the blue-diamonds represent represents the %RMD of subgroup effect

**Fig. 5:**
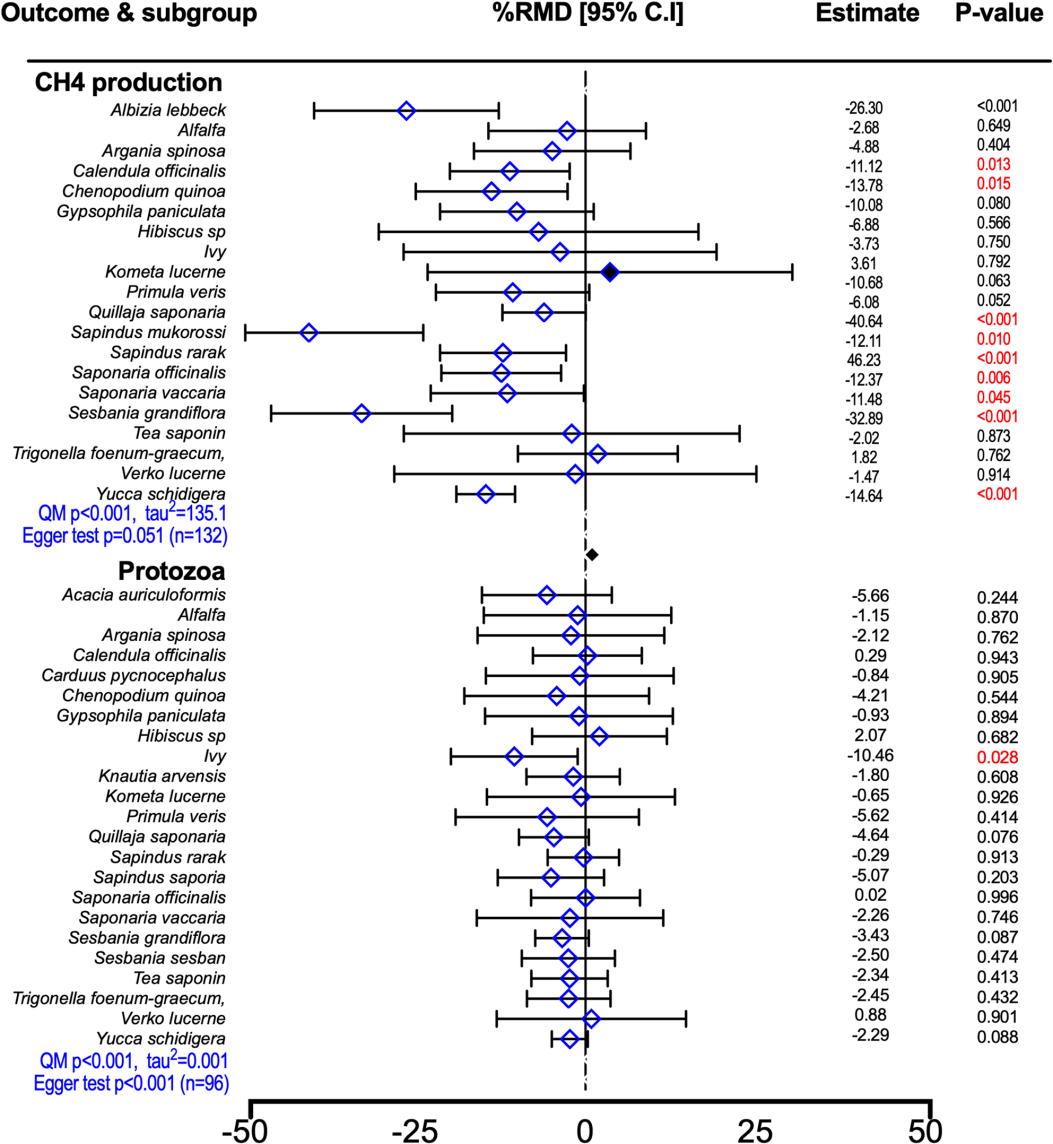
Multilevel subgroup meta-analysis of the effects of saponin sources on methane production and protozoa population. The effect size is expressed at percentage change of weighted raw mean difference (%RMD) at 95% confidence intervals between treatment (saponin extract vs. control substrate). The central-black line represents the zero effect (RMD = 0); the blue-diamonds represent represents the %RMD of subgroup effect

Type of animal donors and in vitro methods (Hohenheim gas test, batch culture, continues culture) were not statistically significant as moderating variables, therefore were disregarded for further evaluation. To evaluate the effects of sources of saponin crude extract, a multilevel mixed model meta-analysis was performed. As shown in the Figs. 2, 3, 4 and 5, there were significant yet divergent effects on in vitro rumen fermentation parameters, with the magnitude and direction of responses partly dependent on the source of saponins. Across all evaluated parameters, the overall subgroup models showed significant effects (*P* < 0.01), except for butyrate concentration, indicating that the sources of saponins extract had different magnitude of effects on rumen fermentation. For DMD and gas production (Fig. 2), different of the origin of saponins extract exhibited as a significant moderator (QM *P* < 0.001). Except for *Kometa lucerne* which enhanced DMD (+ 14.3%; *P* = 0.041), deleterious effects were found with inclusion of saponin crude extract from *Acacia auriculoformis*,* Albizia lebbeck*, Alfalfa, *Quillaja saponaria*,* Sapindus mukorossi*,* Sapindus rarak*, and *Saponaria officinalis* where DMD was reduced between 8.0 and 19.1% (*P* < 0.01). Likewise, gas production was also influenced by the sources of saponin extract (QM *P* < 0.001), although the effect was less pronounced; only *Acacia auriculoformis* reduced gas production (− 7.1%; *P* = 0.003), while other sources showed no significant effect.

For total VFA, the subgroup effect was not significant (*P* = 0.619; Fig. 3), indicating that total VFA responses were relatively consistent across saponin sources. Nevertheless, individual comparisons showed some variation, in which higher VFA concentration was shown by inclusion of saponin from *Calendula officinalis* (+ 20.5%; *P* = 0.013), *Knautia arvensis* (+ 9.2%; *P* = 0.036), *and tea saponin* (+ 28.8%; *P* < 0.001*)* while other sources did not show an effect. In contrast, NH₃ concentration was significantly influenced by saponin source (QM *P* = 0.001). Several sources significantly reduced NH₃ levels such as *Acacia auriculoformis* (-8.1%; *P* = 0.002), *Chenopodium quinoa* (-22.2%; *P* < 0.001), *Gypsophila paniculata* (-18.9%; *P* < 0.001), *ivy* (-57.8%; *P* < 0.001), *Primula veris* (-23.3%; *P* < 0.001), *Quillaja Saponaria* (-9.1%; *P* < 0.001), *Sapindus rarak* (-17.3%; *P* = 0.005), *and Yucca schidigera* (-17.4%; *P* < 0.001). In contrast, supplementing saponin extract from *Verko lucerne* and *Calendula officinalis* resulted in higher(*P* < 0.001) NH_3_-N concentration. The results of analysis of individual VFA profiles demonstrated distinct source-dependent responses (Fig. 4). For acetate, the subgroup effect was significant (QM *P* = 0.01), with some sources decreasing acetate proportion (*P* < 0.05) while others had negligible effects. Propionate also showed significant subgroup effect (QM *P* < 0.001), suggesting that the responses were depended on the saponin sources. Several individual exhibited enhancing effects on propionate concentration including *Sapindus mukorossi*,* Sesbania grandiflora*, and *Yucca schidigera*, ranging between 7.7% and 46.9% (*P* < 0.01). In contrast, butyrate was not affected by saponin source (QM *P* = 0.475).

For CH₄ production (Fig. 5), the sources of saponin extract had different effect to reduced (*P* < 0.001) with many of saponin origins substantially reduced CH_4_ production. The reduction of CH₄ production was highly different, with the greatest reducing effect was shown by *Sapindus mukorossi* (-40.6; *P* < 0 0.001) while the other reduced CH_4_ production between 11 and 26% (Fig. 5), including *Albizia lebbeck*, *Calendula officinalis*,* Chenopodium quinoa*,* Sapindus saporia*,* Saponaria officinalis*,* Saponaria vaccaria*, and *Sesbania grandiflora*. In contrast, protozoa populations were mostly not influenced by saponin sources, except lower protozoal count by Ivy (-10.5%; *P* = 0.028).

### Effects of saponin extract form on rumen fermentation traits, methane output, protozoal abundance, and microbial protein synthesis

As shown in Table 4, comparison of different form of saponin extract (powder vs. liquid) indicated the different responses on rumen fermentation characteristics, CH_4_ output, protozoal abundance, and microbial protein synthesis (*P* ≤ 0.05). Compared to the liquid form, the powder form resulted in a higher rumen pH (*P* = 0.038) and a greater proportion of acetate (*P* = 0.001) but no difference on propionate (*P* = 0.455), leading to a higher acetate-to-propionate ratio (*P* = 0.032). Conversely, the liquid form had higher butyrate (*P* < 0.001) and iso-valerate concentrations (*P* = 0.002). Methane production per unit of substrate also differed between the two forms, with the liquid form producing more CH₄ (mL/g DM substrate) than the powder form (*P* = 0.001). Additionally, protozoal abundance was higher with the powder form (*P* = 0.042), while microbial protein content was greater with the liquid form (*P* = 0.006; Table 4).

**Table 4:** Different saponin extract form on rumen fermentation traits, methane output, protozoal abundance, and microbial protein synthesis

Parameters	pH	C_2_	C_3_	C_4_	IsoC_5_	C_2_:C_3_	CH_4_	Protozoa	MPS
Unit		%	%	%	%	%	mL/g DM	log/mL	mg/dL
N	179	203	198	203	96	210	220	148	32
Form of saponins									
Powder	7.01^a^	64.87^a^	26.31	10.16^b^	2.14^b^	3.07^a^	34.38^b^	5.95^a^	288.1^b^
Liquid	6.44^b^	57.15^b^	23.66	10.92^a^	2.21^a^	2.44^b^	50.05^a^	5.33^b^	426.3^a^
RMSE	0.004	0.221	2.881	0.067	0.024	0.027	0.783	0.014	16.76
p-value	0.038	0.001	0.455	<0.001	0.002	0.032	0.001	0.042	0.006

*C*_2_ acetate, *C*_3_ propionate, *C*_4_ butyrate, *IsoC*_5_ iso-valerate, *C*_2_:C_3_ acetate-to-propionate ratio, *CH*_4_ methane, MPS microbial protein synthesis, *RMSE* root means squared errors

### Meta-regression dose–response relationships and effects of saponin extract forms

**Fig. 6:**
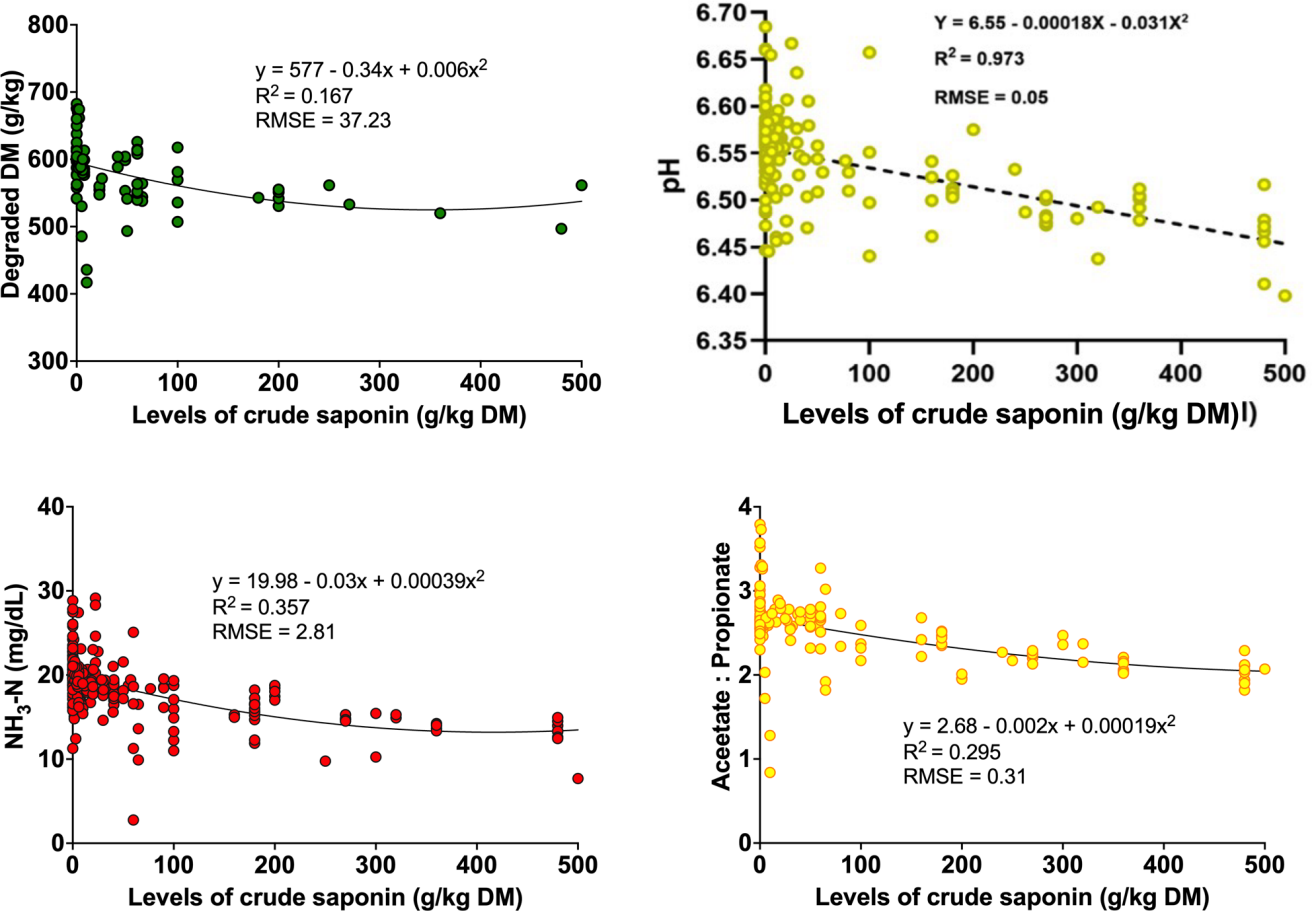
Meta-regression results of the relationship between the levels of saponin crude extract supplementation (g/kg DM) on degraded dry matter (g/kg DM), pH value, NH_3_-N concentration (mg/dL), Acetate to Propionate ratio of in vitro experiments

**Fig. 7:**
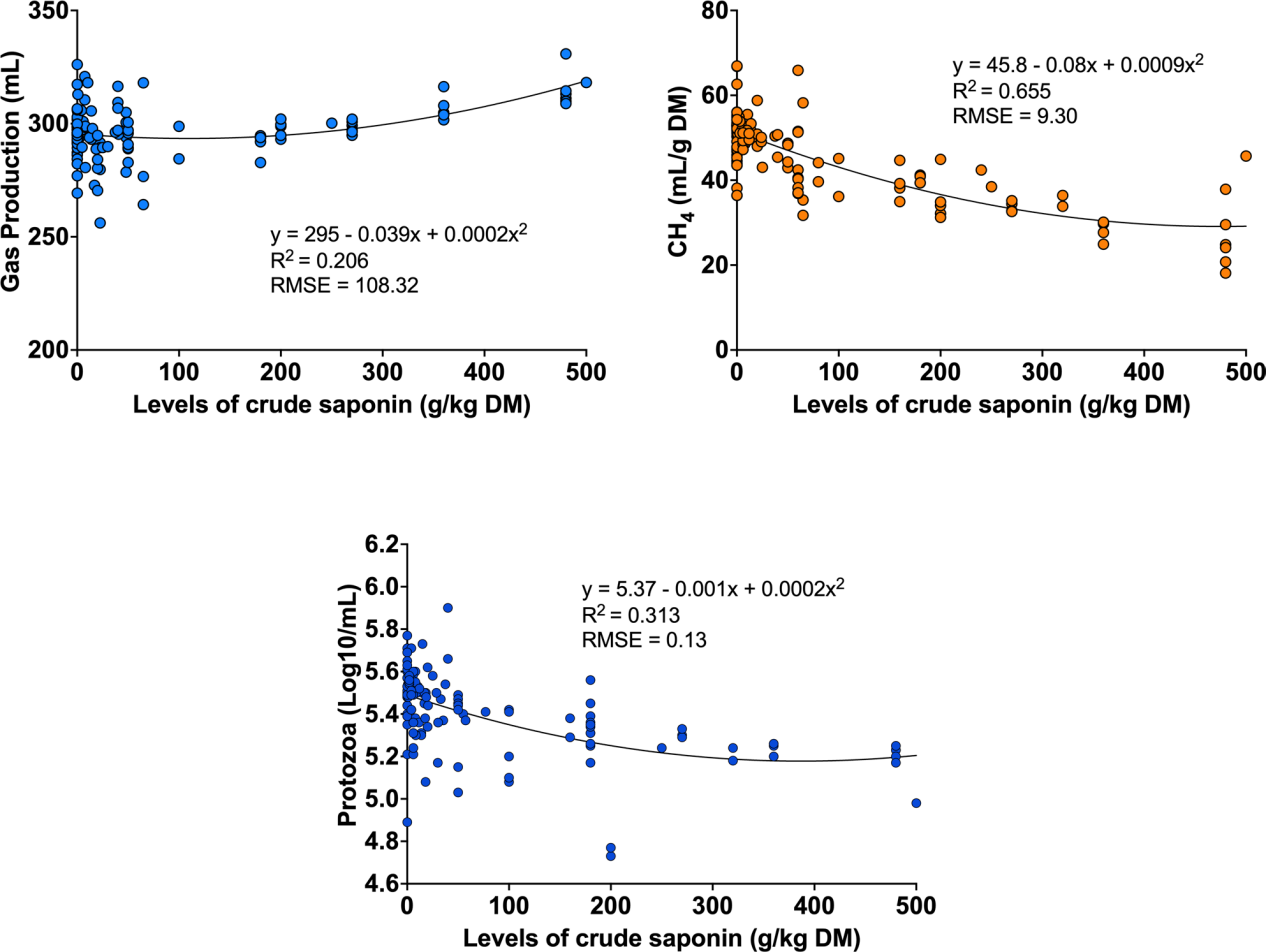
Meta-regression results of the relationship between levels of saponin crude extract supplementation (g/kg DM) on total gas production (mL/g Degraded DM), CH_4_ production (mL/g DM substrates) and protozoa population (log_10_/mL) of in vitro experiments

The dose-response meta-regression plots (Figs. 6 and 7) exhibits non-linear relationships between levels of crude extract saponin supplementation (g/kg DM) and several key in vitro rumen fermentation outcomes. The DMD exhibited a quadratic association (Fig. 6) with levels of saponin extract (R² = 0.167; RMSE = 37.23) with initial decreasing trend in response to increasing dosage, followed by a partial recovery at higher doses. Rumen pH (Fig. 6) showed a consistent, albeit small, downward trend with increasing saponin levels (R² = 0.973; RMSE = 0.05). Concentration of NH_3_-N decreased in a curvilinear manner (Fig. 2C) (R² = 0.357; RMSE = 2.81), indicating the greatest reduction at low-to-moderate doses, with a diminished effect at higher doses. Similarly, the acetate-to-propionate (A: P) ratio declined with increasing levels of saponin extract (Fig. 6) (R² = 0.295; RMSE = 0.31). Gas production responses were also dependent on dosage (Fig. 7). Total gas production per unit of degraded DM followed a quadratic relationship (R² = 0.206; RMSE = 108.32). Methane production per unit substrate decreased with increasing dosage and was best captured by a quadratic model (R² = 0.655; RMSE = 9.30), Protozoal abundance also declined with increasing saponin extract levels (R² = 0.313; RMSE = 0.13).

## Discussion

Saponin extract supplementation has demonstrated a clear, dose-dependent reduction in methanogenesis, accompanied by corresponding changes in ruminal hydrogen dynamics. Specifically, the observed decrease in CH₄ output, alongside a decline in H₂, aligns with the established anti-protozoal activity of saponins [59, 60]. These compounds exert their defaunation effects by disrupting protozoal membranes through binding to cholesterol and other sterol lipids, ultimately leading to membrane deformation and cell lysis [61]. Furthermore, protozoa are closely associated with methanogenic archaea and significantly contribute to hydrogen fermentation; thus, a reduction in protozoal abundance and activity can indirectly diminish the hydrogen supply available for methanogenesis [13, 62]. Particularly, Holotrich protozoa are recognized as major hydrogen producers, underscoring the link between protozoal suppression and reduced CH₄ formation [62, 63].

The suppressed of protozoal counts in this meta-analysis supports a mechanism in which saponins reduce methanogenic activity via pathways associated with protozoa and/or by directly interfering with methanogens, thereby lowering both CH₄ and its essential reductant. Additionally, shifts in CO₂ levels, in conjunction with reduced CH₄ production, indicate changes in electron flow, as methanogenic archaea utilize H₂ as an electron donor to convert CO₂ into CH₄. Notably, no consistent decline in total VFA and no change in total gas production across the dose ranges suggest that CH_4_ reduction primarily occurs through the redirection of metabolic hydrogen and shifts in microbial populations, rather than a disrupted fermentative activity. This finding is consistent with the notion that methanogenesis is only one of several competing sinks for reducing equivalents in the rumen [64, 65].

Moreover, saponin supplementation significantly altered VFA profile, leading to a higher propionate and lower acetate pattern, as well as a reduced acetate-to-propionate ratio. This shift is mechanistically important and is favourable to animal host, given that higher propionate would compete with methanogenesis reducing equivalents. This would decrease the hydrogen pressure that would therefore suppress CH₄ synthesis [66]. Additionally, acetate formation is linked to pathways that generate substrates (CO₂ and reducing equivalents) that support CH₄ production; thus, reduced acetate availability has been associated with lower CH₄ formation [67, 68].

Furthermore, the modest but significant dose-response decline in rumen pH is directionally consistent with these altered fermentation patterns. However, the magnitude of change was small and is unlikely to be biologically limiting under typical in vitro conditions. Simultaneously, the substantial reductions in rumen NH₃-N with increasing saponin levels indicate decreased proteolysis and deamination. This observation aligns with the detergent-like properties of saponins, which tend to inhibit specific rumen microbial groups involved in protein turnover [13, 69]. At high level, this effect can be detrimental because it would lead to lower microbial protein supply to the animals. However, low levels of saponin extract could be beneficial as the effect is negligible, as also supported by no-change of NH_3_-N Fig. 3 when low introduction of saponin extract was carried out. Additionally, reduced urease activity and slower ammonia release have been linked to declines in isoacids and branched-chain VFA, reflecting constrained catabolism of branched-chain amino acids in the rumen [70]. It is also noteworthy that the glyco-fraction of saponins may reduce measurable NH₃ through physical interactions that limit its availability, while decreased protozoal predation can further lower NH₃ generation by diminishing the turnover of microbial protein [13, 61].

It has been suggested that high doses of saponins have been reported to inhibit cellulolytic bacteria and anaerobic fungi, potentially leading to reductions in DMD and OMD [68]. The current synthesis indicates that overall fermentation intensity was not uniformly compromised. For instance, stable VFA production and total gas outputs suggest that saponins may modulate rumen ecology more selectively. This interpretation is further supported by evidence from an in vivo meta-analysis, which demonstrates that increasing saponin supplementation does not necessarily depress nutrient degradation but can instead modulate fibre-degrading communities and rumen fermentation outputs [14]. Even when certain degradability metrics decline at specific dosage ranges, the overall reduction in CH₄ is favorable, as it indicates less energy loss as methane and potentially improved fermentation efficiency [66].

Moreover, beyond dosage effects, the extent of the anti-methanogenic response was influenced by the source and form of the saponin extract. This finding aligns with prior reports indicating that saponin efficacy depends on botanical origin, concentration, and formulation [56, 57, 63]. Historically, *Yucca schidigera* and *Quillaja saponaria* have been widely recognized for their potential in modulating rumen fermentation and methane emissions, further emphasizing the importance of saponin characteristics in determining their effectiveness as nutritional additives.

In addition to the general results of dose-dependent effects, our subgroup meta-analysis demonstrates an important finding where origins of saponin extracts exhibits divergent effects on modulating rumen fermentation and methane production. This may reflect fundamental differences in their chemical structure and biological activity. Earlier, a review by Wina et al. [70] showed diverse phytochemical structures of saponins from different sources (plants) which principally can be classified into glycosylated steroids, triterpenoids, and steroid alkaloids. The different chemical structures of these classes of saponins have been reported to exert distinct sugar moieties, aglycone, and sugar attachment which dictate their biological activities such as their toxicity, antimicrobial effects, and other properties. In our subgroup meta-analysis, for example, rumen modulatory effects were different among saponin sources. Specifically, *Calendula officinalis*,* Knautia arvensis*, and tea (*Camelia sinensis*) exhibits favourable effects to increase VFA production while the other sources showed lack of effects.

Saponins from tea leaves were reported to influence methane production through two main pathways associated with methanogenesis; via direct inhibition of H_2_ production through their antiprotozoal activity and via VFA modulating effects [42, 52]. The suppressing effect of rumen protozoa has been reported to be linearly associated with CH_4_ production in previous meta-analysis [71]. However, our result showed contrary effect regarding the CH_4_ production in which tea saponin had absence reduction effect which might be explained by required longer adaptation to saponin to be able to show antiprotozoal effect. This was supported by the finding from previous in vivo experiment where 3 weeks of tea saponin supplementation did not alter H_2_ production and CH_4_ [52] which is also consistent with other reports in cattle [72, 73]. On the other hand, a comparative study between *Calendula officinalis* and *S. officinalis* [47] showed concurrent increased of VFA and decreased of CH_4_ and protozoa, which support general knowledge on the relationship between protozoa and CH_4_ [71]. However, their methanogenic potential was different primarily due to the levels of saponin extract supplementation rather than their different origins. Interestingly, majority of saponin sources did not influence protozoal counts in this meta-analysis, with evidence of large heterogeneity among studies. the relatively limited and inconsistent effects on protozoa populations in this meta-analysis suggest that antiprotozoal activity is not similar across all saponin sources. This could be attributed to differences in saponin concentration, exposure time (adaptation), or microbial population. As previously mentioned, protozoa are known to adapt to saponins by modifying membrane composition or through symbiotic interactions with bacteria that degrade saponins [74]. The absence of anti-protozoal suppression in some cases suggest the dynamic and adaptive nature of the rumen ecosystem.

Overall, the divergent effect of various saponin sources suggest that rather than acting as a uniform class of phytochemicals, the efficacy of saponins on rumen fermentation and methanogenesis depends on various factors. This includes the dose, adaptation period, sources, basal substrate, and the structure of compounds whose functionality is governed by variations in aglycone type (triterpenoid vs. steroidal), number and linkage of sugar moieties, molecular polarity, and affinity for membrane sterols [59, 70, 74]. These structural features determine how saponins interact with different substrate or diet composition and alter rumen microbiota dynamics, thereby shaping fermentation patterns, nutrient degradation, and gaseous outputs. For instance, a recent study demonstrated that dietary inclusion of tea saponin increased the abundances of fiber-degrading bacteria in the rumen including *Prevotella_1*,* Ruminococcaceae_UCG.002*, and *Prevotellaceae_YAB2003* in soybean hull based diet but not in alfalfa hay diet [75]. Different result on rumen micobial composition was found in in vivo study using tea saponin in which minimal change on rumen microbiota was found [76] which may also explain inconsistency of tea saponin effect in this meta-analysis. Similarly, a study in dairy cow also found no effect of saponin on rumen microbiota [77]. Inconsistency of effects on rumen microbiota from the above studies warrant further investigation regarding the optimal dose, adaptation period, and substrate or diet-specific composition that can optimize the efficacy of saponins.

Several limitations are noted in this meta-analysis especially regarding the potential moderating variables that could not be fully evaluated due to inconsistencies of information in each study. For example, the extraction method of saponins could not be evaluated due to the lack of standardized and consistently reported information across studies, including differences in solvent systems, extraction procedures, and levels of purification. In addition, the inherently heterogeneous nature of saponin extracts, which may vary in composition even within the same plant source depending on extraction conditions, further complicates direct comparisons. As a result, part of the observed variability in fermentation responses may be attributed to differences in extraction methodology rather than solely to the botanical origin of saponins. Future studies should aim to report extraction protocols in detail and consider standardization to enable more precise evaluations.

## Conclusion

In conclusion, our meta-analysis suggests that saponin extracts exert a dose-dependent anti-methanogenic effect by mainly suppressing protozoal count and redirecting hydrogen toward propionate formation. This was supported by reduced CH₄, H₂, and acetate concentration leading to lower acetate: propionate ratio. Saponins also improve ruminal nitrogen utilisation by decreasing NH₃-N without markedly impairing overall fermentation (TVFA and total gas production). However, responses vary depending on the saponin source and formulation. Within the source of saponin extract, inconsistencies among studies were identified, which may depend on specific substrate, microbial adaptation, and dose-specific of saponin-specific sources. Such discrepancies warrant further investigation in vitro and in vivo.

## Data Availability

Dataset is available on request from the authors. The script or code to run the meta-analysis is available in 10.6084/m9.figshare.31939698.
